# Mechano-electrical transduction in trabecular meshwork involves parallel activation of TRPV4 and TREK-1 channels

**DOI:** 10.1080/19336950.2019.1618149

**Published:** 2019-06-04

**Authors:** Oleg Yarishkin, Jackson M. Baumann, David Križaj

**Affiliations:** a Department of Ophthalmology and Visual Sciences, University of Utah, Salt lake City, UT, USA; b Department of Bioengineering, University of Utah, Salt Lake City, UT, USA; c Department of Neurobiology and Anatomy, University of Utah, Salt lake City, UT, USA

**Keywords:** Trabecular meshwork, Mechanotransduction, TRPV4, TREK-1

Intraocular pressure (IOP) controls growth of the eye and maintains its shape. Its amplitude is not fixed - rather, IOP shows time-dependent fluctuations with a periodicity of ~ sec as well as circadian rhythmicity with the highest IOP values occuring at night [1] [2] [3]. Blinking, sneezing, eye rubbing and swimming goggles can elevate IOP from its resting value of 7-15 mm Hg up to 200 mm Hg. Such transient increases in pressure are harmless and perhaps help maintain ocular health but chronic IOP elevations may result in devastating vision loss in a prevalent eye disease, glaucoma. In the healthy primate eye, the primary IOP determinant is the trabecular meshwork (TM) tissue, composed of contractile and mechanosensitive cells that function as gatekeepers of aqueous humor outflow into the canal of Schlemm [4]. IOP increases stimulate TM-intrinsic mechanotransduction (which is currently not well understood) to decrease fluid outflow. In the short term such decreases are compensated but in glaucoma these mechanosensing mechanisms may fail.

IOP can be lowered by genetic and pharmacological targeting of stretch-activated calcium-permeable TRPV4 channels [5]. Because TRPV4 antagonists also stimulate pressure-induced fluid flow in 3D models of trabecular outflow, it is likely that at least a portion of TRPV4 antagonist-dependent IOP decrease is mediated via TM-resident TRPV4 channels. This, however, raises questions about mechanisms that might balance pressure-dependent TRPV4 activation in the healthy eye [6]. A recent study in immortalized TM cells and cells isolated from donor eyes identifies the mechanosensitive TREK-1 (TWIK-related K^+^ isoform 1) channel as a potential candidate for co-regulating TM tensile homeostasis [7].

**Figure 1.**
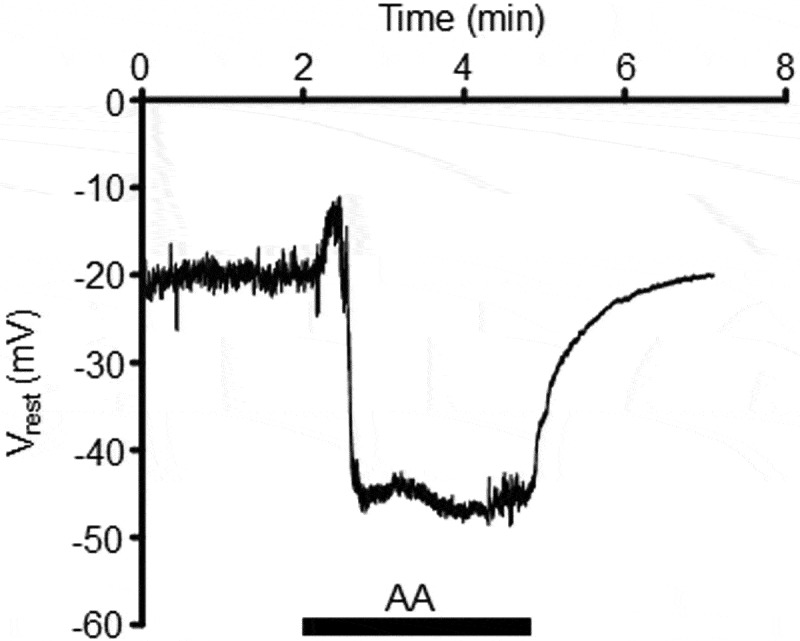
Arachidonic acid induces transient depolarization followed by sustained hyperpolarization of the plasma membrane.

TREK-1 (K2p2.1), encoded by the KCNK2 gene, is composed of tandem pore (P) domains that dimerize to form the K^+^ selectivity filter, an extended extracellular loop near the first transmembrane domain, and intracellular N- and C-termini. Its P_Na_/P_K_ is <0.03 and the channel is widely expressed across stretch-experiencing tissues in the CNS (striatum, hippocampus, olfactory bulb, layer IV cortex) and peripheral tissues (heart, muscle, skin, testes, kidney, gastrointestinal tract) [8]. TREK-1 typically provides a fraction of the background potassium conductance, but Yarishkin et al. [7] found that blockers of voltage-gated (Kv), inwardly-rectifying (Kir), calcium-activated (B_K_/S_K_) do not affect the resting membrane potential (V_rest_) whereas removal of the transmembrane K^+^ gradient and K2p channel blockers (quinine, amlodipine) triggered large and immediate depolarizations. Both primary and immortalized TM cells expressed the transcripts/protein for TREK-1, TASK-1, TWIK-2, and THIK-2 K2p channels yet only TREK-1 shRNA was able to reproduce the effect of K2p blockers on V_rest_, demonstrating that constitutive activation of TREK-1 represents by far the most significant hyperpolarizing component of TM V_rest_. Interestingly, anionic amphipaths (i.e., long-chain polyunsaturated fatty acids) which preferentially insert into the outer leaflet and are believed to convert TREK-1 into a dedicated leak channel [8], induced additional >20 mV hyperpolarizations (Figure 1). Similar hyperpolarizing shift of the PM were also induced by TREK-1 activators (ML-402, ML-335). These results indicate that TM cells possess an additional pool of activatable reserve TREK-1 channels that might endow the cells with autoregulatory response to mechanical stressors.

**Figure 2.**
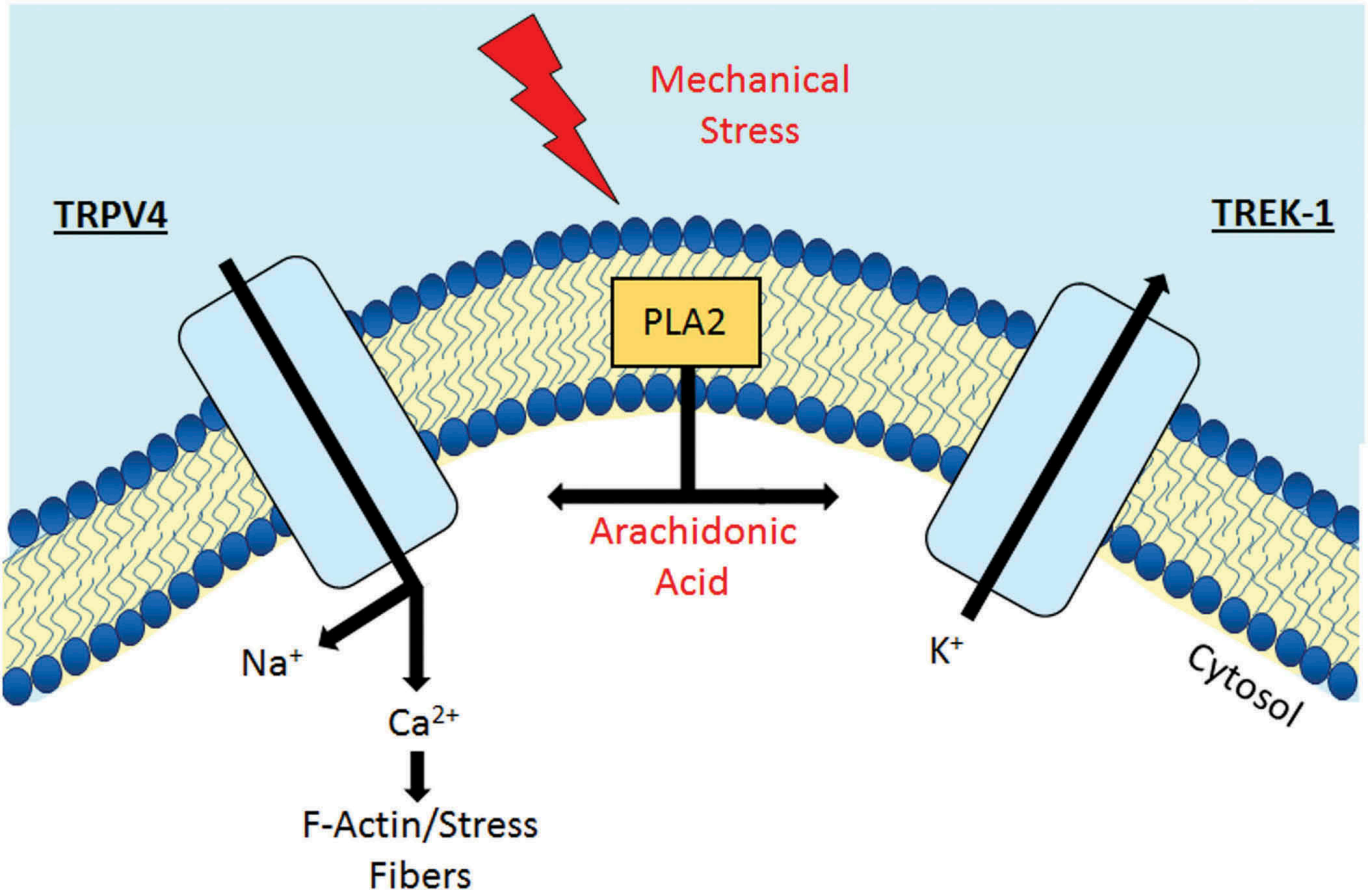
Schematic diagram illustrating a regulatory role of TREK-1 in the TM contractility. The mechanical stress-induced the plasma membrane depolarizing influx of Na^+^ and Ca^2+^ through TRPV4 channels. This TRPV4-mediated hyperpolarization might facilitate Ca^2+^ influx via voltage-gated calcium channels (VOCC) thus increasing the contractility of TM. Besides that, TRPV4-induced elevation of intracellular Ca^2+^ results in augmentation of the TM cells stiffness by strengthening of F-actin stress fibers. Synergistically activated potassium channel TREK-1 facilitates the TM cells relaxation acting as a hyperpolarizing factor that opposes excessive depolarization and limits the activation of VOCC. The activity of both TREK-1 and TRPV4 can be regulated via PLA2 pathways, which involves direct modulation by arachidonic acid and its metabolites.

TREK-1 turned out to mediate the pressure-induced outward current that balances TRPV4 activation. The reversal potential of the pressure-induced current approximated the cells’ resting potential, suggesting that TM mechanosensitivity might be a dynamic function of V_rest_. High-speed pressure clamp, a method that delivers calibrated pressure steps to cells recorded in the whole cell mode, revealed at least two components of the pressure-induced transmembrane current. Consistent with its proposed function as a TM mechanosensor [5], TRPV4 mediated the inward current induced by steps of 15 mm Hg pressure whereas the second component consisted of a large outward current that hyperpolarized the membrane and was largely blocked by K2p antagonists and TREK-1 shRNA. We hypothesize that TREK-1 functions as a voltage and pressure tuner that modulates pressure responsiveness and contractility in part through its control of V_rest_. By opposing the dissipating effect of TRPV4-mediated Na^+^ and Ca^2+^ influx on the membrane potential (Figure 2), TREK-1 might regulate the activation of voltage-operated calcium channels and TM contractility, which in turn could modulate the rate of aqueous outflow [9]. The half-maximal activation of recombinant TREK-1 is ~50 mm Hg [10,11], suggesting that its functionality covers the dynamic range of pressures experienced in the eye. TRPV4 blockers do not affect TM V_rest_, [Ca^2+^]_i_ and resting IOP [12], suggesting that TRPV4 channels do not affect the standing conductance or TM pressure sensing. Both TRPV4 and TREK-1 can be modulated by temperature, pH, phospholipase A2 activation, arachidonic acid, mechanical stressors (shear flow, swelling, strain, compression) and interactions with the extracellular matrix [13,14], suggesting that the TM pressure sensing might be influenced by a multitude of modulating factors that define the set-point of their respective activations.

The possibility that cellular tensional integration involves balancing Ca^2+^, Na^+^, and K^+^ fluxes via parallel activation of at least two distinct mechanotransducers has been explored in small and medium-sized mouse sensory (dorsal root ganglion) neurons, in which TREK1 activation coincides with activity of capsaicin-sensitive TRPV1 channels. Functionally, the two channels may tune the mechanosensitivity of DRG nociceptors: *Trek1^−/−^* mice are more sensitive to mechanical stimulus than wild-type mice [15] whereas *Trpv4*^−/-^ mice appear to be more sensitive to mechanical stressors [16]. Moreover, conditional ablation of TREK-1 from cardiac fibroblasts induced a hypertrophic phenotype that was associated with increased tissue stiffness and heart dysfunction [17]. If this turns out to be the case in the TM, TRPV4 and TREK-1 might collaborate in regulating the adaptive TM response to increased IOP, characterized by increased ECM deposition and contractility in TM myofibroblasts. TRPV4 antagonist and TREK-1 shRNA modulate IOP [5,13] and thus represent potential targets for anti-hypertensive treatments. The results presented by Yarishkin et al. [7] caution, however, that targeting TM mechanotransduction needs to consider the overall tensional homeostasis in these cells.
